# Implementing wait-time reductions under Ontario government benchmarks (Pay-for-Results): a Cluster Randomized Trial of the Effect of a Physician-Nurse Supplementary Triage Assistance team (MDRNSTAT) on emergency department patient wait times

**DOI:** 10.1186/1471-227X-13-17

**Published:** 2013-11-11

**Authors:** Ivy Cheng, Jacques Lee, Nicole Mittmann, Jeffrey Tyberg, Sharon Ramagnano, Alex Kiss, Michael Schull, Fergus Kerr, Merrick Zwarenstein

**Affiliations:** 1 Emergency Services, Sunnybrook Health Sciences Center, Toronto, Canada; 2 Karolinska Institutet, Stockholm, Sweden; 3 Clinical Epidemiology Unit, Sunnybrook Health Sciences Center, Toronto, Canada; 4 HOPE Research Centre, Sunnybrook Health Sciences Centre, Toronto, Canada; 5 Lawrence S. Bloomberg Faculty of Nursing, University of Toronto, Toronto, Canada; 6 Institute of Clinical Evaluative Sciences, Sunnybrook Health Sciences Centre, Toronto, Canada; 7 Department of Emergency Medicine, Austin Health, Heidelberg, Australia; 8 Department of Family Medicine - Schulich School of Medicine and Dentistry, Western University, London, Canada

## Abstract

**Background:**

Internationally, emergency departments are struggling with crowding and its associated morbidity, mortality, and decreased patient and health-care worker satisfaction. The objective was to evaluate the addition of a MDRNSTAT (Physician (MD)-Nurse (RN) Supplementary Team At Triage) on emergency department patient flow and quality of care.

**Methods:**

Pragmatic cluster randomized trial. From 131 weekday shifts (8:00–14:30) during a 26-week period, we randomized 65 days (3173 visits) to the intervention cluster with a MDRNSTAT presence, and 66 days (3163 visits) to the nurse-only triage control cluster. The primary outcome was emergency department length-of-stay (EDLOS) for patients managed and discharged only by the emergency department. Secondary outcomes included EDLOS for patients initially seen by the emergency department, and subsequently consulted and admitted, patients reaching government-mandated thresholds, time to initial physician assessment, left-without being seen rate, time to investigation, and measurement of harm.

**Results:**

The intervention’s median EDLOS for discharged, non-consulted, high acuity patients was 4:05 [95^th%^ CI: 3:58 to 4:15] versus 4:29 [95^th%^ CI: 4:19–4:38] during comparator shifts. The intervention’s median EDLOS for discharged, non-consulted, low acuity patients was 1:55 [95^th%^ CI: 1:48 to 2:05] versus 2:08 [95^th%^ CI: 2:02–2:14]. The intervention’s median physician initial assessment time was 0:55 [95^th%^ CI: 0:53 to 0:58] versus 1:21 [95^th%^ CI: 1:18 to 1:25]. The intervention’s left-without-being-seen rate was 1.5% versus 2.2% for the control (p = 0.06). The MDRNSTAT subgroup analysis resulted in significant decreases in median EDLOS for discharged, non-consulted high (4:01 [95^th%^ CI: 3:43–4:16]) and low acuity patients (1:10 95^th%^ CI: 0:58–1:19]), as well as physician initial assessment time (0:25 [95^th%^ CI: 0:23–0:26]). No patients returned to the emergency department after being discharged by the MDRNSTAT at triage.

**Conclusions:**

The intervention reduced delays and left-without-being-seen rate without increased return visits or jeopardizing urgent care of severely ill patients.

**Trial registration number:**

NCT00991471 ClinicalTrials.gov

## Background

Emergency department (ED) crowding occurs when the demand for emergency services exceeds the ability to provide care in a reasonable amount of time [1]. The main cause of crowding is access block [1-3], where no ward bed is available for patients assessed by the ED requiring admission. Consequently, patients requesting ED care accumulate in the waiting room. In Ontario, discharged patients with longer ED length-of-stay (EDLOS) had higher subsequent hospital admission rates and short-term mortality [4]. Crowding increases both mortality and costs [4-9]. This problem is international [2,5,10,11] in scope and impacts [1,2,5,10-13] governments, [10,14] insurers [7], hospitals, health care workers [11], and patients [4].

In Ontario, there are 163 emergency departments with 5.25 million visits per year [14]. In November 2007, the Ontario Ministry of Health and Long-Term Care declared that reducing EDLOS was a government priority. It established provincial targets, public reporting, and a number of policy initiatives including a Pay-for Results program with financial incentives to reduce EDLOS for targeted hospitals. [15] Our hospital, Sunnybrook Health Sciences Center (Sunnybrook), was one of the targeted hospitals.

The goal of Ontario’s Pay for Results program was for individual hospitals to improve their EDLOS targets by 10% from the previous year (2008) until > =90% of all ED patients reach the set targets [15]. For Sunnybrook, the 2009–10 Pay for Results target was to have > =38% of admitted and > =71% of discharged high acuity patients (Canadian Triage Acuity Scale [CTAS] 1–3) with EDLOS < =8 hrs. For discharged low acuity patients (CTAS 4–5), the Pay for Results target was to have > = 75% of patients with an EDLOS of < =4 hrs. The 2009–10 90^th^ percentile target for physician initial assessment time was set at 5:24, with the future goal of reaching the ideal target of 3:48 [15].

Despite widespread concerns, there are few rigorous evaluations of interventions to reduce EDLOS. The usual triage process has a nurse taking a brief assessment of non-critical patients arriving to the ED and assigning a triage score representing the urgency for emergency physician assessment. Patients who are critically ill (cardiac arrest) bypass triage, and enter a resuscitation room for immediate management. When ED stretchers are full, the rest of the patients, even those with high acuity scores, remain in the waiting room for lengthy periods of time before seeing an emergency physician.

Triage by a nurse alone is intended to prioritize care for the most severely ill patients by assigning acuity scores (CTAS). It is known that correlation of acuity scoring between a triage nurse and physician is poor [16]; therefore, physicians do not triage. The MDRNSTAT (Physician (MD)-Nurse (RN) Supplementary Team At Triage) does not triage. Its role is to interact and start interventions on patients without an ED care space after interacting with the triage nurse. At least 10 studies testing the impact of adding a physician to the existing nurse at triage have shown some benefit [17-26] but only one of these was a randomized trial [20]. We modified an Australian model of physician-triage and report the evaluation of a MDRNSTAT.

The primary objective of this study is to examine the impact and limitations of adding 6.5 hours of MDRNSTAT on EDLOS among non-consulted, discharged patients seen by the emergency physician.

## Methods

### Setting

Sunnybrook is a 1200-bed academic tertiary level hospital in central Toronto. It is a trauma, regional stroke, interventional cardiology, neurosurgical and oncology center. During the study period, Sunnybrook Emergency Department received 45000-patient visits per year with an admission rate of 22%.

### Design

**Figure 1:**
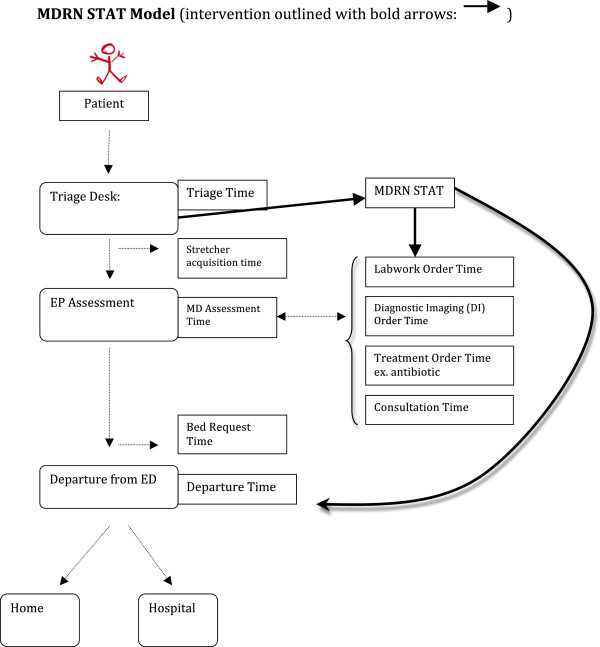
**Pictorial representation of patient management from arrival to discharge in the emergency department.** The control group is outlined with light arrows. The MDRNSTAT intervention is outlined with bold arrows.

We conducted a cluster, randomized-controlled trial of the impact of a Physician-Nurse Supplementary Triage Assessment Team (MDRNSTAT) on EDLOS at Sunnybrook Emergency Department over a 26-week period (October 1, 2009 - April 1, 2010). Clusters were ED patients arriving from 8:00–14:30. During control clusters, ED patients were triaged by a standard nurse for acuity scoring, registered, and assigned an ED stretcher (Figure 1 light arrows). If no stretcher was available, patients stayed in the waiting room until one became available. Afterwards, an emergency physician would assess, order investigations, manage and determine disposition. During intervention clusters, we added a MDRNSTAT without changing time to triage (Figure 1 - bold arrows). Both ambulatory and ambulance patients could be seen by the MDRNSTAT. After being assigned an acuity score by the standard triage nurse, the patient was assessed by the MDRNSTAT physician in a room behind the triage bay where orders were administered instead of waiting for a stretcher in the ED. The MDRNSTAT nurse completed lab and medication orders; whereas, the radiology service completed diagnostic imaging orders. The MDRNSTAT nurse could assist triage or emergency if required. The MDRNSTAT physician could also request consults and, possibly, discharge the patient. If not discharged, the patient would return to the waiting room to wait for a stretcher where the usual ED physician would take over and follow up on the results or consultations. If an ED stretcher was immediately available, patients bypassed the MDRNSTAT and were seen by the usual ED physician.

### Inclusion and exclusion criteria

We included weekday ED patients arriving from 8:00–14:30. We excluded critically ill (CTAS 1) patients requiring immediate resuscitation (i.e. cardiac arrest) since any delays for MDRNSTAT assessment at triage would be unethical and harmful. Patients directed (“directs”) to the ED for an admitting service (eg. oncology) assessment and not an emergency physician, were also excluded from the study.

### Randomization and allocation

Randomization was conducted centrally, before study initiation, using a computer-generated algorithm by the study statistician. The unit of allocation was the ED shift. The randomization schedule ensured 2 control days between interventions to minimize any carryover MDRNSTAT effect. To anticipate predictable surges in the number of ED patients arriving at 11:00, the MDRNSTAT was assigned on weekdays from 8:00–14:30. Our rationale for this timing was to prevent queuing rather than reacting to increased waiting times.

In the daytime weekday study period, there were 65 MDRNSTAT shifts divided amongst 14 physicians and 14 nurses who were currently on staff. There were 66 usual nurse-triage alone (control) shifts. Physicians and nurses voluntarily signed up for paid MDRNSTAT shifts in addition to their regularly scheduled ED shifts. The MDRNSTAT added one physician and one nurse to the 3 physicians and 15–19 nurses typically working each dayshift.

The usual emergency physicians were instructed to not work at triage. The MDRNSTAT could not be blinded to staff; however, patients were unaware of the MDRNSTAT because its schedule was not publicized. Time recordings of primary outcomes were uninfluenced since they were automatically collected by a computerized patient-tracking emergency department information system.

### Data collection procedure

Time data, CTAS levels, and patient disposition were retrieved from two different computerized information systems: Electronic Patient Records and Emergency Department Information Systems. Electronic Patient Records collected diagnostic imaging and laboratory order times. The Emergency Department Information System collected the rest of the data. Ethics approval was obtained from the Sunnybrook Hospital Research Ethics Board (August 2009). The requirement for formal informed consent from patients was waived.

### Outcome measures

The primary cluster outcome measure was median EDLOS for discharged (non-admitted) patients directly seen by emergency physicians (MDRNSTAT or usual ED physician) with no consultation (“non-consulted”) because emergency department interventions could not influence consultation duration or hospital bed availability for admissions. According to the provincial thresholds, EDLOS was the interval between triage time to ED discharge. We analyzed EDLOS according to patient acuity, disposition, and consultation.

Secondary outcome measures were: triage time to start of emergency physician or assessment, EDLOS among patients seen by emergency physician and referred for consultation/admission or left-without-being seen, percentage of CTAS 2–5 patients who were seen within Ontario’s Pay for Results wait-time target thresholds, and left-without-being-seen rate, a widely used measure of safety and satisfaction. [2,5,27,28] Other secondary outcome measures were triage time to: laboratory, diagnostic imaging, consultation, and bed request order time.

To measure unintended harm, we searched our database for patients who returned to our hospital within 48 hours after being discharged from triage by MDRNSTAT. These patients’ charts were reviewed (IC) for a change in management, admission or death. Management change occurred if diagnosis or treatment was different between the first and second visit. No time parameters or external hospital data could be collected for patients who left-without-being-seen. All outcome measures were assigned a priori.

### Sample size and statistical analysis

We estimated that a sample size of 50 clusters (shifts) per group, with 32 individuals per cluster, would have greater than 90% power to detect a difference of 30 minutes between the group medians. We used 30 minutes as a clinically significant reduction, because previous trials determined this difference was associated with decreased 7-day mortality, re-admission of discharged patients [4] and left-without-being seen rate [27]. The Ontario Ministry of Health’s target was a 10% reduction [14] of Sunnybrook’s baseline physician initial assessment time of 324 minutes, which also equals 30 minutes. We used a standard deviation of 70 minutes, intra-cluster correlation of 0.1 [29] using a two-sided t-test, and a significance level of 0.05.

The analyses were carried out comparing patients in groups, control versus treatment, adjusting for the clusters (shifts) in which the patients appeared. This resulted in 66 control clusters (for a total of 3163 visits) compared to 65 intervention clusters (for a total of 3137 visits) and therefore the visits were not treated as independent observations for analysis purposes. The number of clusters was increased to 65 in order to compensate for the variable number of individuals per cluster.

Descriptive statistics were calculated for variables of interest with categorical measures summarized using counts and percentages. The primary outcome, EDLOS, as well as the other secondary time variables were summarized using medians. For analysis purposes, the outcomes were log transformed and the mean group differences of EP’s, MDRNSTAT, the combination of the two and the control group were compared using linear models adjusting for the correlation among observation taken from the same cluster (shift). Confidence intervals for medians were determined with 1000 bootstrap simulations. The 2.5^th^ and 97.5^th^ percentile respectively were reported as the 95% confidence interval. For variables with no events, such as rate of harm we calculated one-sided 95% confidence intervals using the Hanley estimate of 3/n where n is the total sample size of interest [30]. All analyses were carried out using SAS Version 9.1 (SAS Institute, Cary, NC, USA).

## Results

### Participants

**Figure 2:**
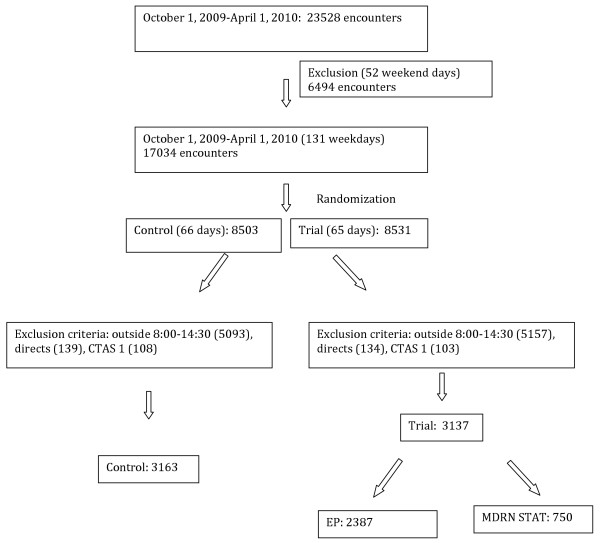
Randomization and encounter allocation with associated inclusion and exclusion criteria.

There were 17,034 weekday emergency department visits (8531 randomized to intervention and 8503 to control) during the study. After excluding patients arriving outside 8:00–14:30, critically ill, or “directs”, there were 66 control clusters (3163 visits) group and 65 intervention clusters (3137 visits). Of the 3137 intervention cluster patients, 750 (24%) patients waiting for an ED stretcher were initially seen by the MDRNSTAT. The regularly scheduled emergency physician (EP) solely managed the remaining 2387 patients (Figure 2) only after the patient obtained a stretcher. The two study groups were similar with respect to baseline characteristics of sex, age, and CTAS (Table 1). The percentages of discharged patients were the same in each group (79.1%).

**Table 1:** **Characteristics of MDRNSTAT Trial and Control Clusters from October 2009-April 2010 (EP** = **Emergency Physician, MDRNSTAT** = **Physician-Nurse Supplementary Team at Triage)**

	**Control Days**		**Trial Days**						
			**EP**		**MDRNSTAT**		**Combined (EP + MDRNSTAT)**		***p***
	**No.**	**%**	**No.**	**%**	**No.**	**%**	**No.**	**%**	
No. of Patients (randomization)	8503	36.1					8531	36.3	
No. of Patients (8:00–14:30)	3163	37.2	2387		750		3137	36.5	*0.58*
Visits/shift	47.9				48.3				
No. of Clusters (Shifts)	66				65				
No. of Patients seen after 8:00 arriving before 8:00	331	31.2	295		78		373	32.5	*0.55*
Sex (Male)	1389	43.9					1362	43.4	*0.71*
Age (mean)	56yo		56yo		54yo		56yo		
CTAS 1 (of 8:00–14:30)	108	2.9	97		6		103	3.1	*0.84*
CTAS 2–3 (of 8:00–14:30)	2489	78.7	1828	76.6	615	82.0	2443	77.9	*0.45*
CTAS 4–5 (of 8:00–14:30)	674	21.3	559	23.4	135	18.0	694	22.1	*0.45*
Directs (of 8:00–14:30)	139	4.1	117		17		134	4.0	*0.85*
Admits (of 8:00–14:30)	586	18.5	451	18.9	154	20.5	605	19.3	*0.44*
Discharges (of 8:00–14:30)	2503	79.1					2482	79.1	*0.99*
No. of CT/Study Discharges	280	11.2					295	11.9	*0.44*
No. of CT/Study Admissions	160	27.3					163	26.9	*0.89*
No. of CT/Study Discharges & Admissions	440	14.2					458	14.8	*0.51*

The combined (MDRNSTAT + EP) and MDRNSTAT group had improved primary outcomes. For the combined group, EDLOS was significantly reduced by 24’ for non-consulted higher acuity patients (CTAS 2–3) (p = .005) but was not significant for discharged, non-consulted lower acuity patients (CTAS 4–5). The MDRNSTAT significantly reduced the discharged, non-consulted lower acuity EDLOS by 56’ (p < .0001) (Table 2).

**Table 2:** Discharged EDLOS (Consulted and Non-Consulted), Admission EDLOS, Physician Initial Assessment, LWBS and Mortality

	**Control**	**Trial**		
		**EP**	**MDRNSTAT**	**Combined (EP + MDRNSTAT)**
**Discharges LOS – Not Consulted**				
CTAS 2–3 *n*	1547	1156	379	1535
LOS (95^t%^ CI)	4:29 (4:19–4:38)	4:07 (3:59–4:18)	4:01 (3:43–4:16)	4:05 (3:58–4:15)
		*p* = *.01, k* = *0.1*	*p* = *.03, k* = *0.1*	*p* = *.005, k* = *0.1*
Pay for Results Threshold (71%)	86.0%	89.1%	89.2%	89.1%
		*p* = *.02*	*p* = *.13*	*p* = *.01*
CTAS 4–5 *n*	614	497	126	623
LOS (95^%^ CI)	2:06 (2:02–2:14)	2:08 (2:01–2:21)	1:10 (0:58–1:19)	1:55 (1:48–2:05)
		*p* = *0.74*	*p* <*.0001, k* = *0.1*	*p* = *.12*
Pay for Results Threshold (75%)	84.4%	81.1%	92.1%	83.3%
		*p* = *.17*	*p* = *.04*	*p* = *.67*
**Discharges LOS - Consulted**				
CTAS 2–3 *n*	305	205	81	286
LOS	7:19	7:06	6:25	6:48
		*p* = *0.5*	*p* = *0.1*	*p* = *.27*
Pay for Results Threshold (71%)	57.0%	61.5%	64.2%	62.2%
		*p* = *.37*	*p* = *.30*	*p* = *.23*
CTAS 4–5 *n*	37	32	6	38
LOS	4:57	4:40	4:19	4:40
		*p* = *.71*	*p* = *.95*	*p* = *.73*
Pay for Results Threshold (75%)	40.5%	34.4%	50%	36.8%
		*p* = *.78*	*p* = *1.0*	*p* = *.93*
**Admissions LOS**				
Admissions *n*	586	451	154	605
LOS	12:03	11:41	11:20	11:36
		*p* = *0.24*	*p* = *0.1*	*p* = *.21*
Pay for Results Threshold (38%)	21.3%	22.6%	25.3%	23.3%
		*p* = *.67*	*p* = *0.34*	*p* = *0.34*
**Physician Initial Assessment**				
*n*	3092/3163	2341/2387	750	3091/3137
Pay for Results 90^th^ percentile (3:48) (95^th^ CI)	4:25 (4:11–4:36)	3:57 (3:47–4:08)	1:08 (1:01–1:14)	3:31 (3:22–3:42)
Median PIA (95^th^ CI)	1:21 (1:18–1:25)	1:13 (1:10–1:16)	0:25 (0:23–0:26)	0:55 (0:53–0:58)
		*p* = *.0005*	*p* <*.0001*	*p* <*.0001*
**LWBS**				
*n*	69/3163	44/2387	4/750	48/3137
Percentage	2.2%	1.9%	0.53%	1.5%
		*p* = *0.43*	*p* = *.001*	*p* = *.06*
**Mortality**				
*n*	5/3163	2/2387	0/750	2/3137
Percentage	0.16%	0.08%	0%	0.06%
		*p* = *.71*	*p* = *.59*	*p* = *.45*

For the secondary outcomes among non-consulted CTAS 2–3 patients, the combined group met its 10% improvement target, and came close to the ideal threshold of 90%, with 89.1% of combined patients reaching the Pay for Results EDLOS target of < =8 h. For non-consulted CTAS 4–5 patients, the combined group reached the 10% improvement threshold, but did not exceed the ideal target (83.3%); however, the MDRNSTAT did (92.1%).

For patients who required admission or consultation, the intervention made little difference.

The combined group decreased physician initial assessment time by 26-minutes with a 90^th^ percentile of 3:31. The MDRNSTAT decreased physician initial assessment time by 56-minutes with a 90^th^ percentile of 1:08. The ideal provincial 90^th^ percentile physician initial assessment time threshold is 3:48. In 2007–8, Sunnybrook hospital’s 90^th^ percentile physician initial assessment time was 5:42.

The combined group and MDRNSTAT reduced the left-without-being-seen rate (Table 2).

Some of the secondary outcomes including laboratory, diagnostic imaging, and consultation times, for the control and trial groups improved (Table 3). For discharges and admissions, the combined group had shorter ordering times for all the three modalities than control. For admissions, MDRNSTAT hospital bed request time was faster than control. For discharges, the combined group ordered less bloodwork and imaging than control.

**Table 3:** Bloodwork, diagnostic imaging, and consultation request times for discharges and admissions; bed request and wait for bed times for admissions

	**Control**	**Trial**		
		**EP**	**MDRNSTAT**	**Combined (EP + MDRNSTAT)**
**Discharges:**				
*n*	2503	1890	592	2482
% Bloodwork (BW) Request	37.0%	32.9%	43.8%	35.5%
% Imaging (DI) Request	51.0%	45.8%	61.8%	49.6%
% Discharged	79.1%	79.2%	78.9%	79.1%
% Consulted	13.7%	12.5%	14.7%	13.1%
*Median Times:*				
BW Request Time	1:47	1:42	1:02	1:32
DI Request Time (95^%^ CI)	2:16 (2:08–2:24)	1:56 (1:48–2:04)	0:51 (0:43–0:57)	1:38 (1:32–1:46)
		*p* = *.0025*	*p* <*.0001*	*p* <*.0001*
Consult Request Time	3:20	2:59	2:40	2:54
		*p* = *.08*	*p* = *.02*	*p* = *.01*
**Admissions:**				
*n*	586	451	154	605
% Bloodwork (BW) Request	80.9%	83.4%	79.2%	82.3%
% Imaging (DI) Request	84.5%	83.8%	88.3%	85.0%
% Admitted	18.5%	18.9%	20.5%	19.3%
*Median Times:*				
BW Request Time	1:38	1:51	1:07	1:39
DI Request Time	2:41	2:43	1:05	2:11
		*p* = *.87*	*p* <*.0001*	*p* = *.0027*
Consult Request Time (95^%^ CI)	3:57 (3:44–4:08)	3:49 (3:37–4:10)	3:36 (3:16–4:02)	3:46 (3:36–4:03)
		*p* = *.80*	*p* = *0.17*	*p* = *.36*
Bed Request Time	7:41	7:30	7:01	7:19
		*p* = *.50*	*p* = *.02*	*p* = *.19*
Wait for Bed Time	2:58	3:14	2:59	3:10

*BW*: Bloodwork.

*CTAS*: Canadian Triage Acuity Scale.

*CT*: Computer Tomography Scan.

*CI*: Confidence interval.

*DI*: Diagnostic Imaging.

*LOS*: Length of Stay.

*LWBS*: Left without Being Seen.

The MDRNSTAT discharged 26.1% (196/750) of patients from triage. Only 3 of these patients returned to the ED within 48 hours. None of these patients were admitted or died. Their charts were reviewed and were found to have the same discharge diagnoses between the first and second visit: staple reassessment, urinary tract infection and social concerns. All 3 patients were discharged on the second visit without change in management or treatment. None met our definition of harm. The Hanley 95% CI estimate [30] for harmful events was 0–1.53%.

## Discussion

This was a pragmatic study. Because the only changes were the added MDRNSTAT resource, its benefits could be applied to other hospitals similar to Sunnybrook.

This study had a number of limitations. It is a single-center study, and one of two randomized control trials [20]. To increase its generalizability from academic tertiary level centers, a multi-center trial is recommended. Coverage on weekdays from 8:00–14:30 meant that patients arriving on weekends and evenings could not benefit. Focusing MDRNSTAT shifts during times of crowding or adaptively engaging MDRNSTAT when bed blockage occurs may benefit from future study.

Because physicians and nurses entered time data, measurement bias was possible. This study was not blinded, but all health care workers were under the same Pay for Performance program. Additionally, the health care workers who volunteered to be MDRNSTAT also worked in the department as control or part of the intervention on different days. Consequently, all physicians would have the same pressure to discharge patients within targets. However, wait times could have been shortened because the MDRNSTAT wanted to discharge patients quicker, or faster physicians/nurses volunteered to be the MDRNSTAT. The differences between the MDRNSTAT and the control could be attributed to patient selection; however, time gains were observed with the combined group as well.

It could be argued that the benefits of this trial arose from the extra staff, and would have occurred had they been deployed in the ED rather than at triage. However, Ontario hospitals run lean with fewer ward beds per capita than many US settings [12,31] creating access block. Consequently, ED admissions can occupy ED stretchers for a median 10 hours creating a bottleneck of physical space for assessment. Under these conditions, added staff capacity would be optimized at triage since patients do not have to wait for an available ED stretcher in order to see a physician. To further explore this issue, future studies could compare a control physician-nurse team in the department to the MDRNSTAT.

We only reviewed Sunnybrook hospital’s database for harm. Patients returning to a different hospital, or dying outside of the hospital would have been missed. However, distribution should have been similar in both arms given randomization. This suggests no detectable harm, but it is important to bear in mind that this study was not powered to detect mortality differences.

For patients managed solely by the ED, MDRNSTAT significantly reduced EDLOS and physician initial assessment time. The MDRNSTAT initiated actions (laboratory, diagnostic imaging, and consultation request times) that shortened emergency physician decisions and patient delays. Because waiting times to disposition were reduced, left-without-being-seen rates declined, resulting in greater patient throughput.

For patients whose EDLOS was dependent upon factors external to the ED (i.e. consultation), the gains with shortened physician initial assessment and request times were diluted by the time required to complete these external requests by non-ED hospital staff.

Serious illnesses seen in ED’s, such as ischemia, have time-dependant treatments [32]. Skilled, timely care [10,33] is crucial; hence the focus of performance indicators on waiting times [34]. However, rules focusing solely on wait-times, without thoughtful improvement in care processes may compromise quality of care [35] and increase costs [36]. The 4-hrs to disposition rule in the UK was estimated to cost an extra £820 million without consistent health improvement [36]. None of 750 patients assessed in this prospective, randomized-controlled trial experienced harm according to our measures; suggesting that this method to speed critical decisions does not compromise quality of care.

Emergency departments have successfully implemented fast-track areas. [37,38]. However, the MDRNSTAT can see mixed acuity patients, whereas the fast-track population will be mainly ambulatory, lower acuity patients. While fast-track might be useful in community hospitals, Sunnybrook has few (27%) CTAS 4–5 patients compared to the provincial average (48.2%) [39].

Some have argued that it is inappropriate to have the “the ED compensate(s) for deficiencies in the larger organization by subordinating its own needs and priorities” [40]. The ED is squeezed between rising demand for emergency access to beds and increasing block as hospitals beds are closed or occupied by more lucrative, elective patients. MDRNSTAT cannot address systemic emergency department [41] or health economic problems [42]. Thus, we support the conclusion of multiple studies that emphasize the need for “throughput-output” solutions, such as hospital bed availability [11,43,44], long-term care, diagnostic imaging, and associated funding. However, the MDRNSTAT may provide an additional solution to those requiring government co-operation or hospital resources [8,45-47].

## Conclusion

The MDRNSTAT was effective in decreasing EDLOS, physician initial assessment time and left-without-being-seen rates of discharged patients requiring ED services in an urban, academic tertiary-level hospital, without compromising the quality of patient care. Given that the risk of admission and death for ED patients is associated with increasing EDLOS [4], the time gains associated with MDRNSTAT could improve patient outcomes. We are currently analyzing data on the economic value of the MDRNSTAT program.

## Competing interests

The authors declare that there have no competing interests.

## Authors’ contributions

IC: public responsibility for the whole content, conception and design, acquisition of data, analysis and interpretation of data, drafting of the manuscript, critical revision of the manuscript, statistical analysis, obtaining funding, administrative, technical, material support. IC had full access to all of the data in the study and takes responsibility for the integrity of the data and the accuracy of the data analysis. MZ: public responsibility for part of the content, conception and design, interpretation of data, drafting of the manuscript, critical revision of the manuscript for important intellectual contact, statistical analysis supervision. JT: public responsibility for part of the content, conception, critical revision of the manuscript for important intellectual content, obtaining funding, administrative support. JL: public responsibility for part of the content, conception and design, interpretation of data, critical revision of the manuscript, supervision. NM: public responsibility for part of the content, design, critical revision of the manuscript, supervision. SR: public responsibility for part of the content, acquisition of data, critical revision of the manuscript, technical/material support. AK: public responsibility for part of the content, design, analysis of data, statistical analysis, critical revision of the manuscript. MS: public responsibility for part of the content, design, critical revision of the manuscript, supervision. FK: public responsibility for part of the content, conception and design, critical revision of the manuscript, technical support. All authors read and approved the final manuscript.

## Pre-publication history

The pre-publication history for this paper can be accessed here:

http://www.biomedcentral.com/1471-227X/13/17/prepub

## References

[B1] Bond K Frequency, determinants and impact of overcrowding in emergency departments in Canada: a national survey Healthc Q 2007 10 4 32 40 10.12927/hcq.2007.19312 18019897

[B2] Forero R Access block and ED overcrowding Emerg Med Australas 2010 22 2 119 135 10.1111/j.1742-6723.2010.01270.x 20534047

[B3] Forster AJ The effect of hospital occupancy on emergency department length of stay and patient disposition Acad Emerg Med 2003 10 2 127 133 10.1111/j.1553-2712.2003.tb00029.x 12574009

[B4] Guttmann A Association between waiting times and short term mortality and hospital admission after departure from emergency department: population based cohort study from Ontario Canada. BMJ 2011 342 d2983 10.1136/bmj.d2983 PMC3106148 21632665

[B5] Bernstein SL The effect of emergency department crowding on clinically oriented outcomes Acad Emerg Med 2009 16 1 1 10 10.1111/j.1553-2712.2008.00295.x 19007346

[B6] Johnson KD Winkelman C The effect of emergency department crowding on patient outcomes: a literature review Adv Emerg Nurs J 2011 33 1 39 54 10.1097/TME.0b013e318207e86a 21317697

[B7] Krochmal P Riley TA Increased health care costs associated with ED overcrowding Am J Emerg Med 1994 12 3 265 266 10.1016/0735-6757(94)90135-X 8179727

[B8] Maa J The waits that matter N Engl J Med 2011 364 24 2279 2281 10.1056/NEJMp1101882 21675887

[B9] Shen YC Hsia RY Association between ambulance diversion and survival among patients with acute myocardial infarction JAMA 2011 305 23 2440 2447 10.1001/jama.2011.811 21666277 PMC4109302

[B10] Hughes G Four Hour Target for EDs: The UK Experience Emerg Med Australas 2010 22 5 368 373 10.1111/j.1742-6723.2010.01326.x 21040479

[B11] Hoot NR Aronsky D Systematic review of emergency department crowding: causes, effects, and solutions Ann Emerg Med 2008 52 2 126 136 10.1016/j.annemergmed.2008.03.014 18433933 PMC7340358

[B12] Position Statement on Emergency Department Overcrowding 2009 http://caep.ca/sites/default/files/caep/files/edoc_position_statement_board_approved_june_2009_gl.pdf

[B13] Mitka M Emergency department overcrowding gives ambulances the runaround JAMA 2006 295 13 1504 1505 16595747 10.1001/jama.295.13.1504

[B14] Ontario Tackles ER Times with $109 Million Investmenthttp://www.health.gov.on.ca/en/public/programs/waittimes/strategy.aspx

[B15] Ontario Wait-Time Strategyhttp://www.health.gov.on.ca/en/public/programs/waittimes/

[B16] Brillman JC Does a physician visual assessment change triage? Am J Emerg Med 1997 15 1 29 33 10.1016/S0735-6757(97)90043-7 9002565

[B17] Choi YF Wong TW Lau CC Triage rapid initial assessment by doctor (TRIAD) improves waiting time and processing time of the emergency department Emerg Med J 2006 23 4 262 265 discussion 262–5 10.1136/emj.2005.025254 16549569 PMC2579496

[B18] Grant S Spain D Green D Rapid assessment team reduces waiting time Emerg Med 1999 11 2 72 77 10.1046/j.1442-2026.1999.00017.x

[B19] Han JH The effect of physician triage on emergency department length of stay J Emerg Med 2010 39 2 227 233 10.1016/j.jemermed.2008.10.006 19168306

[B20] Holroyd BR Impact of a triage liaison physician on emergency department overcrowding and throughput: a randomized controlled trial Acad Emerg Med 2007 14 8 702 708 10.1111/j.1553-2712.2007.tb01864.x 17656607

[B21] Partovi SN Faculty triage shortens emergency department length of stay Acad Emerg Med 2001 8 10 990 995 10.1111/j.1553-2712.2001.tb01099.x 11581086

[B22] Redmond AD Buxton N Consultant triage of minor cases in an accident and emergency department Arch Emerg Med 1993 10 4 328 330 10.1136/emj.10.4.328 8110326 PMC1286043

[B23] Richardson JR Braitberg G Yeoh MJ Multidisciplinary assessment at triage: a new way forward Emerg Med Australas 2004 16 1 41 46 10.1111/j.1742-6723.2004.00541.x 15239754

[B24] Subash F Team triage improves emergency department efficiency Emerg Med J 2004 21 5 542 544 10.1136/emj.2002.003665 15333524 PMC1726448

[B25] Terris J Making an IMPACT on emergency department flow: improving patient processing assisted by consultant at triage Emerg Med J 2004 21 5 537 541 10.1136/emj.2002.003913 15333523 PMC1726434

[B26] Travers JP Lee FCY Avoiding prolonged waiting time during busy periods in the emergency department: is there a role for the senior emergency physician in triage? Eur J Emerg Med 2006 13 6 342 17091056 10.1097/01.mej.0000224425.36444.50

[B27] Mohsin M A population follow-up study of patients who left an emergency department without being seen by a medical officer Emerg Med J 2007 24 3 175 179 10.1136/emj.2006.038679 17351221 PMC2660023

[B28] Baker DW Stevens CD Brook RH Patients who leave a public hospital emergency department without being seen by a physician. causes and consequences JAMA 1991 266 8 1085 1090 10.1001/jama.1991.03470080055029 1865540

[B29] Turner RM Thompson SG Spiegelhalter DJ Prior distributions for the intracluster correlation coefficient, based on multiple previous estimates, and their application in cluster randomized trials Clin Trials 2005 2 2 108 118 10.1191/1740774505cn072oa 16279132

[B30] Hanley JA Lippman-Hand A If nothing goes wrong, is everything all right? Interpreting zero numerators JAMA 1983 249 13 1743 1745 10.1001/jama.1983.03330370053031 6827763

[B31] No Vacancy Ontario Health Coalition Finds Hospital Overcrowding at Untenable Levelshttp://www.ontariohealthcoalition.ca

[B32] Terkelsen CJ System delay and mortality among patients with STEMI treated with primary percutaneous coronary intervention JAMA 2010 304 7 763 771 10.1001/jama.2010.1139 20716739

[B33] McCarthy S ACEM position on a time-based access target in Australian and New Zealand EDs Emerg Med Australas 2010 22 5 379 383 10.1111/j.1742-6723.2010.01328.x 21040481

[B34] Schull M Development of a Consensus on Evidence-Based Quality of Care Indicators for Canadian Emergency Departments. ICES Investigative Report 2010 Toronto: Institute for Clinical Evaluative Sciences

[B35] The Healthcare Commission Investigation into Mid Staffordshire NHS Foundation Trust 2009 London, Bristol, Nottingham, Leeds, Manchester, and Solihull: Commission for Health Care Audit and Inspection

[B36] Jones P Schimanski K The four hour target to reduce Emergency Department ‘waiting time’: a systematic review of clinical outcomes Emerg Med Australas 2010 22 5 391 398 10.1111/j.1742-6723.2010.01330.x 20880296

[B37] Hooker RS Klocko DJ Larkin GL Physician assistants in emergency medicine: the impact of their role Acad Emerg Med 2011 18 1 72 77 10.1111/j.1553-2712.2010.00953.x 21166731

[B38] Schneider SM The future of emergency medicine Acad Emerg Med 2010 17 9 998 1003 10.1111/j.1553-2712.2010.00854.x 20836784

[B39] NACRS Emergency Department Visits by Triage Level and Age Group 2009 http://www.cihi.ca/cihi-ext-portal/internet/en/document/types+of+care/hospital+care/emergency+care/HP17_Query_Info_Rev_01

[B40] Wears RL Cook RI Getting better at being worse Ann Emerg Med 2010 56 5 465 467 10.1016/j.annemergmed.2010.08.002 20864211

[B41] Hsia RY Kellermann AL Shen YC Factors associated with closures of emergency departments in the United States JAMA 2011 305 19 1978 1985 10.1001/jama.2011.620 21586713 PMC4063529

[B42] Mitka M Economics may play role in crowding, boarding in emergency departments JAMA 2008 300 23 2714 2715 10.1001/jama.2008.753 19088342

[B43] Khare RK Adding more beds to the emergency department or reducing admitted patient boarding times: which has a more significant influence on emergency department congestion? Ann Emerg Med 2009 53 5 575 585 10.1016/j.annemergmed.2008.07.009 18783852

[B44] Steele R Kiss A EMDOC (Emergency Department overcrowding) Internet-based safety net research J Emerg Med 2008 35 1 101 107 10.1016/j.jemermed.2007.03.022 17976788

[B45] Kellermann AL Martinez R The ER, 50 years on N Engl J Med 2011 364 24 2278 2279 10.1056/NEJMp1101544 21675886

[B46] Tang N Trends and characteristics of US emergency department visits, 1997–2007 JAMA 2010 304 6 664 670 10.1001/jama.2010.1112 20699458 PMC3123697

[B47] Hampton T Experts predict visits by baby boomers will soon strain emergency departments JAMA 2008 299 22 2613 2614 10.1001/jama.299.22.2613 18544716

